# circAFF1 Aggravates Vascular Endothelial Cell Dysfunction Mediated by miR-516b/SAV1/YAP1 Axis

**DOI:** 10.3389/fphys.2020.00899

**Published:** 2020-08-05

**Authors:** Hong-guang Wang, Hua Yan, Chen Wang, Mi-mi Li, Xin-ze Lv, Hai-dong Wu, Zhan-hai Fang, Dong-li Mo, Zhi-yuan Zhang, Bin Liang, Ke-guan Lai, Jing-yu Bao, Xue-jia Yang, Hong-juan Zhao, Shuang Chen, Yi-mu Fan, Xiao-guang Tong

**Affiliations:** ^1^College of Pharmacy, Nankai University, Tianjin, China; ^2^Department of Neurosurgery, Tianjin Huanhu Hospital, Tianjin, China; ^3^Department of Neurology, Tianjin Huanhu Hospital, Tianjin Key Laboratory of Cerebral Vascular and Neurodegenerative Diseases, Tianjin, China; ^4^Tianjin Institute, of Neurosurgery, Tianjin Huanhu Hospital, Tianjin, China; ^5^Tianjin Key Laboratory of Early Druggability Evaluation of Innovative Drugs, Tianjin International Joint Academy of Biomedicine, Tianjin, China; ^6^Drug Safety Evaluation Center, Tianjin International Joint Academy of Biomedicine, Tianjin, China; ^7^Department of Neurosurgery, People’s Hospital of Ningxia Hui Autonomous Region, Yinchuan, China; ^8^Department of Respiratory Medicine, Songjiang Sijing Hospital, Shanghai, China

**Keywords:** vascular endothelial cell, subarachnoid hemorrhage, hypoxic, circAFF1, YAP1

## Abstract

Pathological vascular endothelial damage caused by hypoxia is the basis of many vascular-related diseases. However, the role of circular RNA in hypoxic vascular injury is still poorly understood. Here, we found that hypoxia induced AFF1 circular RNA (circAFF1) can activate the SAV1/YAP1 and lead to the dysfunction of vascular endothelial cells. In HUV-EC-C and HBEC-5i cells, circAFF1 was upregulated under CoCl_2_ induced hypoxic conditions. The abnormal expression of circAFF1 inhibited the proliferation, tube formation, migration of vascular endothelial cells. The effect of circAFF1 is achieved by the adsorption of miR-516b to release SAV1, which in turn causes the phosphorylation of YAP1. Moreover, we found that the upregulation of circAFF1 in 235 Patients with subarachnoid hemorrhage. Taken together, we clarify the role of circAFF1/miR-516b/SAV1/YAP1 axis in vascular endothelial dysfunction and its potential early diagnostic value of disease caused by hypoxia injury in blood vessels.

## Introduction

Ischemia occurs when local tissues cannot meet their energy needs, and hypoxia is an important component of ischemic injury (Eltzschig et al., 2014). Hypoxia plays an important role in protective and pathological vascular adaptation. Hypoxia causes damage to vascular endothelial cells, and endothelial dysfunction disrupts blood flow (Mao et al., 2013; Giannarelli et al., 2014; Zhao et al., 2015). The occurrence and development of vascular endothelial injury is an important cause of many diseases, such as cardiovascular and cerebrovascular diseases, diabetes and tumors (Shan et al., 2017; Qureshi-Baig et al., 2019). Insufficient nutrient supply and hypoxia can cause myocardial infarction and stroke (Lim, 2017; Brait et al., 2019). While, bleeding can also seriously threaten human health, such as subarachnoid hemorrhage. However, whether vascular dysfunction will accelerate the development of these diseases and how to achieve early diagnosis and prevention has received little attention.

Circular RNA (circRNA) is a class of covalent closed-loop RNA without a 5′ cap and 3′ polyadenylation tail (Salzman, 2016; Kristensen et al., 2019). Previous studies have found thousands of circRNAs in different species. CircRNA has multiple biological functions, it can restore the expression of downstream genes by adsorbing miRNA, stabilize protein complexes, and even encode proteins (Schneider and Bindereif, 2017; Vo et al., 2019; Zheng et al., 2019; Dragomir et al., 2020). For instance, CircEPSTI1 is highly expressed in triple-negative breast cancer and promotes the malignant progression of breast cancer by simultaneously adsorbing miR-4753 and miR-6809 (Chen et al., 2018). The expression of RNA circSKA3 can stabilize the binding of Tks5 and integrin β1 to promote breast cancer progression (Du et al., 2020). In myoblasts, circ-ZNF609 can be translated into proteins in a splice-dependent and cap-dependent manner to participate in the regulation of myoblast proliferation (Legnini et al., 2017). Recently, several circRNAs have been analyzed to be related to hypoxia (Boeckel et al., 2015). However, the biological functions and clinical significance of circRNAs implicated in Vascular endothelium remain largely unknown.

The present study shows that circAFF1 was significantly up-regulated in vascular endothelial cells under hypoxic conditions induced by CoCl_2_ and causes vascular endothelial cells to malfunction. Mechanistically, circAFF1 promotes SAV1 expression by adsorbing miR-516b, leading to activation of the Yes-associated protein 1 (YAP1), which causes vascular endothelial cells to malfunction. In addition, abnormal upregulation of circAFF1 was also found in SAH patients. These results suggest that occurrence of SAH is correlated to the functional injury of vascular endothelial cells and circular RNA may be a potential diagnostic marker and target for relative disease treatment.

## Materials and Methods

### Clinical Sample Collection and RNA Extraction

A total of 235 patients enrolled between August 2018 to January 2020 at Tianjin Huanhu Hospital were selected to analyze the expression of circAFF1. The patients were divided into five groups according to Hunt-Hess classification. Written, informed consent was obtained from the individuals for the publication of any potentially identifiable images or data included in this article. After blood samples were collected from SAH patients, serum was prepared within 2 h by centrifugation at 1200 g for 10 min. Total RNA was isolated by using TRIzol reagent (Invitrogen, United States) in accordance with the manufacturer’s instructions. The absorbance values of all RNA samples at A260 and A280 were tested to identify the quality of RNA.

### Cell Culture and Treatments

Human endothelial cell lines HUV-EC-C (from umbilical vein endothelium) and HBEC-5i (from cerebral microvascular endothelium) were obtained from American Type Culture Collection (ATCC, United States) and cultured in DMEM (Gibco) supplemented with 10% FBS (Gibco, United States), 30 μg/ml endothelial cell growth supplement (E0760, Sigma) and 1% penicillin–streptomycin solution (Gibco) under hypoxic (300 μM CoCl_2_ for 10 h) or normoxic conditions. The cells were maintained in a humidified atmosphere incubator with 5% CO_2_ at 37°C and passaged regularly.

### qRT-PCR

Genomic DNA was isolated with MiniBEST Universal Genomic DNA Extraction Kit Ver.5.0 (Takara, Japan), and total RNA was extracted from cells using TRIzol reagent (Invitrogen, United States) in accordance with the manufacturer’s instructions. For circRNA detection, before RNase R treatment, RNA was divided into two equal parts. one of which was treated with RNase R to obtain circRNA, and the other was used to detect the expression of GAPDH. RNase R (Epicenter Technologies, United States) was used to remove linear RNA. 2 (μg of total RNA was treated with or without RNase R (3 U/(μg) at 37 ((°C for 30 min, then the treated RNA was purified with the RNeasy MinElute Cleanup Kit (Qiagen, Germany). The fractions were extracted using a PARIS^TM^(^TM^ Kit (Life Technologies, United StatesUSA) in accordance with the manufacturer’s protocol. Complementary DNAs (cDNA) were synthesized from total RNA using the PrimeScript RT Reagent Kit (Tiangen), and QPCR was carried out on a LightCycler480 sequence detector (Roche, Switzerland), along with a Fast SYBR Green Master Mix kit (Tiangen), in accordance with the manufacturer’s recommendation. GAPDH and U6 were used as internal controls. The results were then analyzed with the 2-(Δ(Δ C t relative expression method. All primer sequences were as followed: circAFF1-F: 5′-CTTGAAAGTGCCTGCCAAAG-3′, circAFF1-R: 5′-GGCTTCCTGGTTGCGTCT-3′; AFF1-F: 5′-CAAC ATTAGCCACAATCCA-3′, AFF1-R: 5′-AATCTGTCACCGAA GCAC-3′; GAPDH-F: 5′-GACCTGACCTGCCGTCTA-3′, GAPDH-R: 5′-AGGAGTGGGTGTCGCTGT-3′; U6-RT: 5′-GTCGTATCCAGTGCAGGGTCCGAGGTGCACTGGATACGA CAAAATATGGAAC-3′, U6-F: 5′-TGCGGGTGCTCGCTTCG GCAGC-3′, U6-R: 5′-CCAGTGCAGGGTCCGAGGT-3′; miR-516b-RT: 5′-GTCGTATCCAGTGCAGGGTCCGAGGTGCACTG GATACGACAAAGTG-3′, miR-516b-F: 5′-TGCGGATCTGGA GGTAAGAAG-3′, miR-516b-R: 5′-CCAGTGCAGGGTCCGAG GT-3′.

### Oligonucleotide Transfection

Small interfering RNA (siRNA), miRNA mimics, miRNA inhibitors, and negative control oligos were obtained from GenePharma (Shanghai, China). All those were transfected into cells by using Lipofectamine 8000 (Invitrogen). circAFF1 siRNA: 5′-AGUCAGUUGAGUUU GTACAAUTT-3′; scramble siRNA: 5′-UUGCUAAGCGUCGGUCAAUTT-3′.

### circRNA Plasmid Construction and Transfection

CircAFF1 was synthesized and cloned into the plenti-ciR-GFP-T2A vector (IGE Biotech Co, China) to construct circAFF1 overexpression plasmids. The plasmids were detected by sequencing. Then the plasmids were transfected into HEK293T cells to package lentivirus. After circAFF1 lentivirus infection, HUV-EC-C and HBEC-5i cells were treated with 2 μg/mL puromycin for 3 days and screened to obtain cell lines with high circAFF1 level.

### Cell Viability Assay

Cell Counting Kit-8 (CCK-8) (Dojindo Laboratories, Tokyo, Japan) was used to measure cell viability. Cells were seeded in 96-well plates at a final concentration of 5 × 10^3^/well. When the cell density is about 70%, cells were transfected and cultured at 37°C with 5% CO2. After incubated with 10 (μL of CCK-8 solution at 37°C for 4 h, Optical density (OD) values at 490 nm were obtained using a microplate reader (Multiskan MK, Thermo Fisher Scientific Inc.; United States).

### Tube Formation Assay

The pre-chilled DMEM medium and Matrigel (BD Biocoat, United States) were mixed in an equal volume and added to a 48-well plate at a volume of 200 μl per well, and subsequently placed in humidified atmosphere incubator with 5% CO_2_ at 37°C for 30 min. The treated cells were then seeded into Matrigel-coated 48-well plates at a concentration of 2 × 10^5^ per well. After continuous cultivation at 37°C with 5% CO_2_ for 24 h, images of the tube were taken under a light microscope (Nikon, Japan). The tube structures were assessed to evaluate the ability of tube formation. We performed quantitative analysis of the obtained images using the angiogenesis analysis function of Image J software.

### Wound Healing Assay

The treated cells were seeded into 24-well plates at a density of approximately 50% cells were maintained in a humidified atmosphere incubator with 5% CO2 at 37°C. 24 h later, use the 200 μL pipette tips head to make a straight scratch in the middle of the well. The cells were washed with PBS and then continue to grow in humidified atmosphere incubator. After 48 h, cells were washes with PBS for three times and migration was photographed with a light microscope (Nikon, Japan) and the distance was measured and normalized to the 0 h control as the relative migration rate for comparison.

### Cell Apoptosis Assay

Cell apoptosis was analyzed using the Annexin V-FITC/propidium iodide (PI) detection kit (Beyotime, Shanghai, China). Cells were plated in 6-well dishes at a density of 2 × 10^5^/well. After treated, adjusted the cell concentration to 106 cells/ml, then centrifuge 200 μl cells at 1000 rpm (4°C) for 5min. After washing twice with pre-chilled PBS, centrifuge again at 1000 rpm × 5 min (4°C). Subsequently, collected cells were incubated in 400 μL of binding buffer with 5 μL of Annexin V-FITC and 5 μL of PI in the dark for 30 min at room temperature and analyzed with a flow cytometer (Millipore).

### Western Blot Analysis

Proteins were extracted using RIPA lysis buffer supplemented with 1% proteinase inhibitor and quantified by a BCA kit (Thermo, United States). Harvest proteins were then separated by 10% sodium dodecyl sulphate–polyacrylamide gel electrophoresis (SDS-PAGE) and transferred onto PVDF membranes (Millipore, United States). After blocking by using 5% BSA, the membranes were incubated overnight at 4° (°C with the following specific primary antibodies (anti-GAPDH, anti-p84, anti-SAV1, anti-YAP1, and anti-p-YAP1). After washing, the membranes were incubated for 2 h with secondary HRP antibodies (Sigma-Aldrich). Protein levels were detected using enhanced ECLprime (GE Healthcare) and captured and analyzed using a Las-3000luminescent Image Analyzer (Fuji Film).

### Biotin-Coupled Probe Pull-Down Assay

In order to obtain the miRNA bound by circAFF1, the biotinylated circAFF1 probe and oligo probe (GenePharma, China) were incubated with M-280 streptavidin magnetic beads (Invitrogen, United States) for 2 h at room temperature to generate probe-coated beads. Then, approximately 1 × 10^7^ cells were harvested, and the lysate was incubated with probe-coated beads at 4°C overnight. After washing, the RNAs were eluted and extracted with RNeasy Mini Kit (Qiagen). The expressions of miRNAs were then analyzed by qRT-PCR assay.

### Fluorescence *in situ* Hybridization (FISH)

A Cy3-labeled circAFF1 probe and a Cy5-labeled miRNA-516b probe were designed and synthesized by GenePharma (Shanghai, China). After the cells were fixed with 4% paraformaldehyde and treated with Triton-X100, the fluorescently labeled probes were incubated with the cells. After washing with 2 × SSC buffer, DAPI was added to stain the nuclear DNA. The signals of the probe were detected by the Fluorescent *In Situ* Hybridization Kit (GenePharma, China) in accordance with the manufacturer’s protocols. All images were acquired on Nikon A1 Confocal Microscope system (Nikon, Japan).

### Luciferase Reporter Assay

HEK293T cells were seeded into 96-well plates and cultured for 24 h. The synthesized circAFF1 sequence containing wild-type or mutated miR-516b binding sites (CTCCAGA) was cloned into the dual luciferase reporter vector. Wild and mutant fluorescent reporter plasmids were co-transfected with miR-516b mimics in HER293 cells. After transfection, cells were cultured for another 48 h. Transactivation assays were performed with Dual-Luciferase Assay System (Promega), and luciferase activities were detected using the Luminoskan Ascent reader system (Thermo Scientific, United States).

### Exosome Extraction, Purification, and Quantification

Cells were harvested and centrifuged at 1000 × *g* for 30 min to remove cell debris. The supernatants were then subjected to 300 kDa molecular weight cut off to concentrate and washed with PBS. Then the concentrated samples were ultracentrifugation at 100,000 × *g*, 4°C on a 5.5% sucrose pad for 15 h. Harvest exosomes were re-suspended in PBS and quantified prior to further analysis.

### Statistical Analysis

Statistical analyses were performed using SPSS 17.0 (SPSS, Inc., Chicago, IL, United States). Multi-group analysis of variances was carried out by one-way ANOVA test followed by post-hoc tests. The significance of differences between two groups was examined using Student’s *t*-test. All experiments were repeated at least four times, and the data are expressed as mean ± SD. Statistical significance was considered at *P* < 0.05 (^∗^*P* < 0.05 or ^∗∗^*P* < 0.01, respectively, in the figures).

## Results

### Cytoplasm-Localized circAFF1 Is Upregulated in Vascular Endothelial Cells Under CoCl_2_ Treatment

**FIGURE 1:**
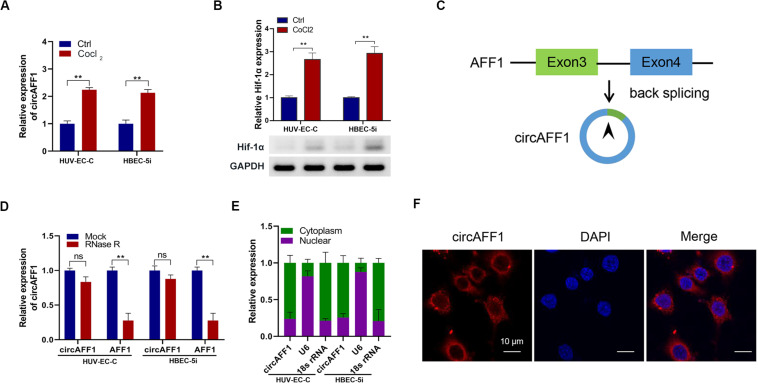
Validation of circAFF1 in endothelial cells under CoCl_2_ treatment. **(A)** Relative expression of circAFF1 in HUV-EC-C and HBEC-5i cells under CoCl_2_ treatment. **(B)** Relative expression of AFF1 in HUV-EC-C and HBEC-5i cells under CoCl_2_ treatment. **(C)** CircAFF1 derived from back-splicing of exons 3 and 4 of the AFF1 gene. **(D)** qRT-PCR analysis of circAFF1 and AFF1 mRNA after treatment with or without RNase R in HUV-EC-C and HBEC-5i cells. **(E)** qRT-PCR analysis of circAFF1 using nuclear and cytoplasmic fractions of HUV-EC-C and HBEC-5i cells. **(F)** FISH confirmed that circAFF1 was predominantly located in the cytoplasm. Nuclei were stained with DAPI, and circAFF1 was labeled with Cy3. Data are presented as means of three experiments, and error bars represent SD (**P < 0.01).

We treated vascular endothelial cells HUV-EC-C and HBEC-5i with CoCl_2_ and found that circAFF1 was upregulated in both cell lines (Figure 1A). The up-regulation of Hif-1α expression after CoCl_2_ treatment indicates that CoCl_2_ can simulate cell hypoxia (Figure 1B). Subsequently, we searched circAFF1 on the circBase^1^ and found that circAFF1 was derived from exons 3 and 4 of the AFF1 gene (Figure 1C). In order to confirm the correctness of circAFF1, we used RNase R to detect the expression of circAFF1 and total AFF1, respectively. The results show that our target circAFF1 can resist RNase R treatment (Figure 1D). In order to analyze the cellular localization of circAFF1, we used both qRT-PCR and immunofluorescence methods for verification. We found that circAFF1 was enriched in the cytoplasm fraction and mainly distributed in the cytoplasm (Figures 1E,F). Taken together, these results indicated that circAFF1 was upregulated in the endothelial cells under hypoxic conditions and was predominantly localized in the cytoplasm.

### CircAFF1 Inhibits the Proliferation, Tube Formation, Migration and Contributes to Apoptosis of Endothelial Cells *in vitro*

**FIGURE 2:**
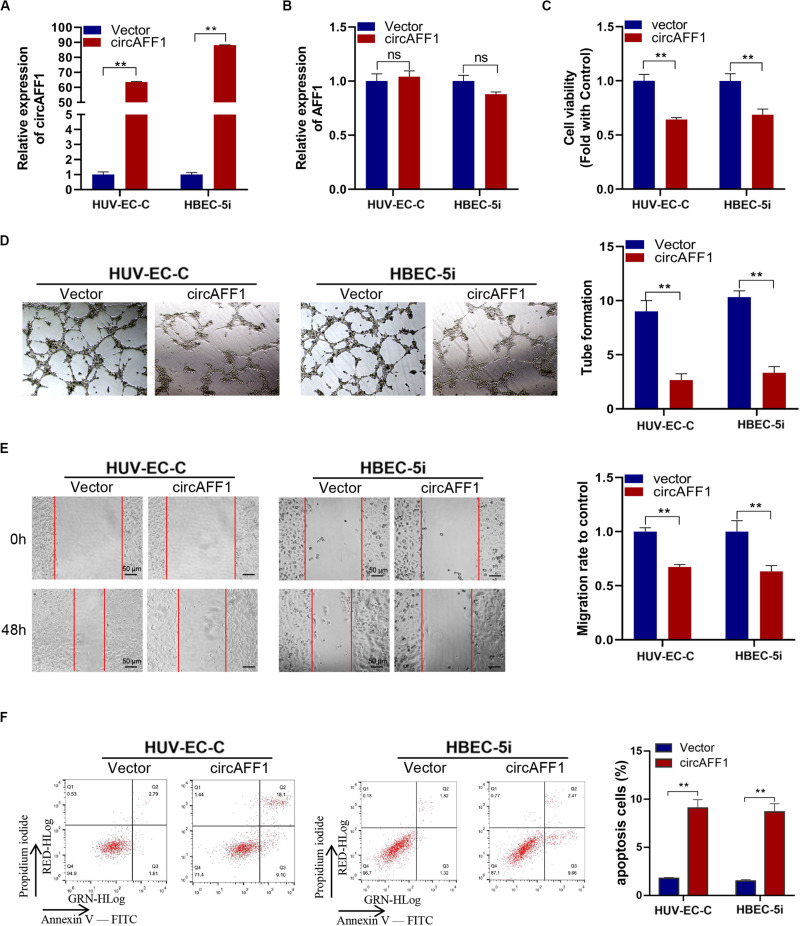
Overexpression of circAFF1 inhibited the proliferation and tube formation and promotes the apoptosis of endothelial cells *in vitro*. qRT-PCR analysis of circAFF1 **(A)** and AFF1 mRNA **(B)** in HUV-EC-C and HBEC-5i cells after stable transfection of circAFF1 or vector. **(C)** Cell viability of HUV-EC-C and HBEC-5i cells transfected with circAFF1 or vector was evaluated by CCK-8 assays. **(D)** Tube formation capability of HUV-EC-C and HBEC-5i cells transfected with circAFF1 or vector was evaluated. **(E)** Migration capability of HUV-EC-C and HBEC-5i cells transfected with circAFF1 or vector was evaluated by wound healing assays. **(F)** Apoptosis capability of HUV-EC-C and HBEC-5i cells transfected with circAFF1 or vector was evaluated by apoptosis assay. Data are presented as means of three experiments, and error bars represent SD (**P < 0.01).

We established circAFF1 stably overexpressing cell lines via transfecting with the circAFF1 vector to investigate the potential biological effect of circAFF1 on HUV-EC-C and HBEC-5i cells. The overexpression efficiencies of circAFF1 were detected by qRT-PCR (Figure 2A), and the expression level of AFF1 was not affected by circAFF1 changes (Figure 2B). Cell viability assay, tube formation assay, wound healing assay and cell apoptosis assay showed that overexpression of circAFF1 inhibited the viability (Figure 2C), tube formation (Figure 2D), migration (Figure 2E) and promoted the apoptosis (Figure 2F) of HUV-EC-C and HBEC-5i cells. These results suggested that circAFF1 is a negative regulator of vascular endothelial cells.

### Knockdown of circAFF1 Can Counteract the Effects of Hypoxia on Vascular Endothelial Cells

**FIGURE 3:**
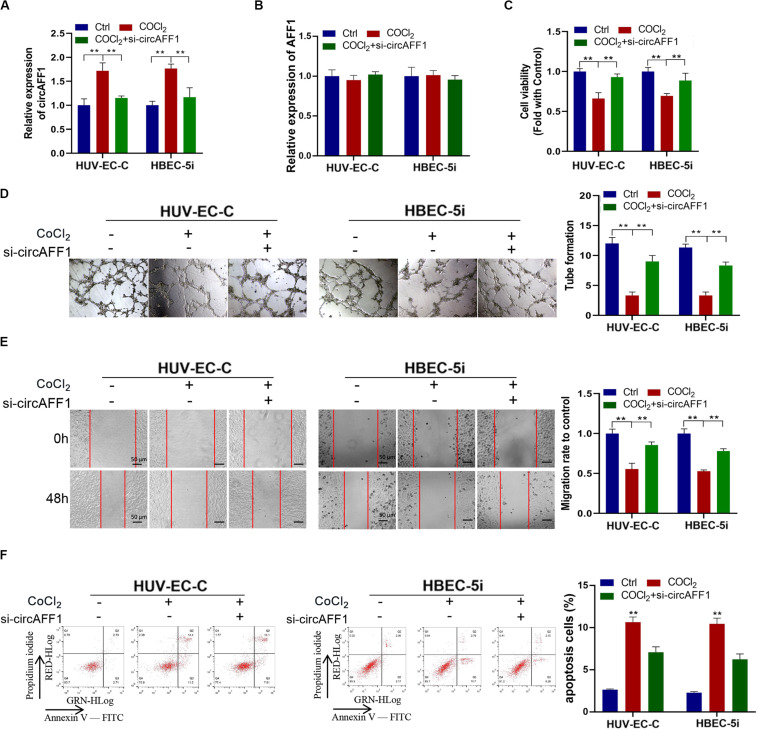
Under CoCl_2_ treatment, circAFF1 inhibited the proliferation and tube formation and promotes the apoptosis of endothelial cells *in vitro*. **(A,B)** Under CoCl_2_ treatment, qRT-PCR analysis of circAFF1 and AFF1 mRNA levels in HUV-EC-C and HBEC-5i cells after silencing circAFF1. **(C)** Under CoCl_2_ treatment, the cell viability of HUV-EC-C and HBEC-5i cells after silencing circAFF1 was evaluated by CCK-8 assays. **(D)** The tube formation capability of HUV-EC-C and HBEC-5i cells after silencing circAFF1 was evaluated. **(E)** The migration capability of HUV-EC-C and HBEC-5i cells after silencing circAFF1 under hypoxic conditions was evaluated by wound healing assays. **(F)** Under hypoxic conditions, the apoptosis capability of HUV-EC-C and HBEC-5i cells after silencing circAFF1 was evaluated by apoptosis assay. Data are presented as means of three experiments, and error bars represent SD (**P < 0.01).

We silenced the expression of circAFF1 in HUV-EC-C and HBEC-5i cells by using RNA interference to verify the biological effect of circAFF1 on endothelial cells under hypoxic conditions. The expression of circAFF1 was detected by qRT-PCR analysis, and the result confirmed that circAFF1 expression was higher in the HUV-EC-C and HBEC-5i cells under hypoxic conditions and decreased when circAFF1 was silenced (Figure 3A). While, circAFF1 siRNA did not cause changes in the amount of AFF1 mRNA (Figure 3B). Cell viability assay, tube formation assay, migration and cell apoptosis assay also demonstrated that silencing circAFF1 can promote the proliferation (Figure 3C), tube formation (Figure 3D), migration (Figure 3E) and inhibit the apoptosis (Figure 3F) of HUV-EC-C and HBEC-5i cells *in vitro* under hypoxic conditions. These all verified that Knockdown of circAFF1 can partially restore hypoxia-induced inhibition of endothelial cell function.

### CircAFF1 Serves as a miRNA Sponge for miR-516b

**FIGURE 4:**
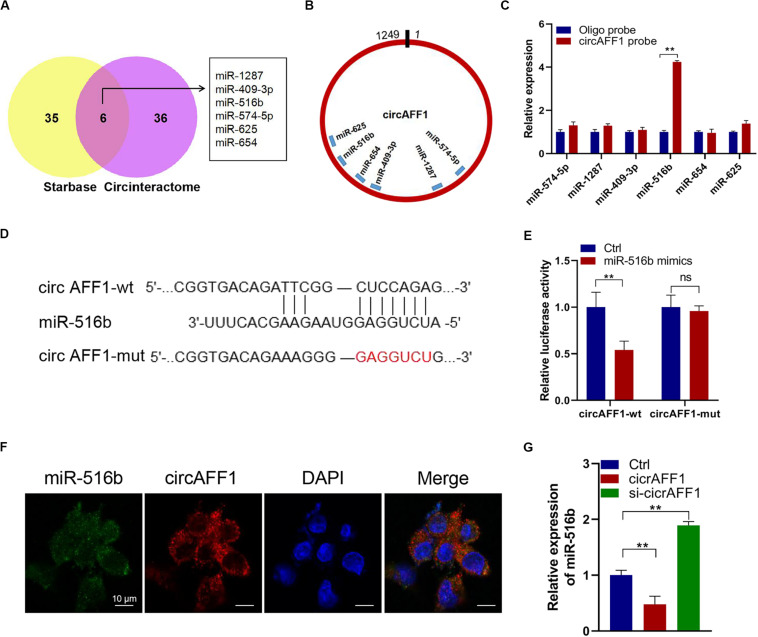
CircAFF1 served as a miR-516b sponge in endothelial cells. **(A)** Six potential target miRNAs of circAFF1 were predicted by CircInteractome and Starbase. **(B)** Schematic model showed the putative binding sites of six miRNA candidates associated with circAFF1. **(C)** Relative levels of six miRNAs in HUV-EC-C lysates pulled down by circAFF1 probe or oligo probe. **(D)** Schematic of circAFF1 wild-type (wt) and mutant (mut) luciferase reporter vectors. **(E)** Luciferase reporter assay in HEK293T cells co-transfected with miRNA mimics, circAFF1-wt, or circAFF1-mut plasmids. **(F)** FISH showed the colocalization between circAFF1 and miR-516b in HUV-EC-Cs. Nuclei were stained with DAPI. CircAFF1 was labeled with Cy3,and miR-516b was labeled with Cy5. **(G)** qRT-PCR analysis of miR-516b in HUV-EC-Cs after transfected with circAFF1or silencing circAFF1. Data are presented as means of three experiments, and error bars represent SD (**P < 0.01).

We further explored whether circAFF1 can bind to miRNAs to evaluate the potential mechanisms of circAFF1 in endothelial cells. We found six potential target miRNAs (miR-1287, miR-409-3p, miR-516b, miR-574-5p, miR-625, and miR-654) of circAFF1 by CircInteractome and Starbase (Figure 4A). We also presumed the binding sites of these candidate miRNAs in circAFF1 (Figure 4B). Subsequently, the miRNAs pulled down by biotinylated probes were purified and analyzed by qRT-PCR. Among these candidate miRNAs, only miR-516b could be strikingly pulled down by the circAFF1 probe in both HUV-EC-C (Figure 4C). To detect the binding of circAFF1 and miR-516b, wild and mutated binding site luciferase reporter plasmid was used (Figure 4D). Results showed that miR-516b upregulation inhibited the relative luciferase activity of circAFF1-WT, but not circAFF1-mut. This suggesting that miR-516b can interact with circAFF1 (Figure 4E). Moreover, FISH analysis in endothelial cells also displayed that circAFF1 and miR-516b were co-localized in the cytoplasm (Figure 4F). More importantly, our data also revealed that overexpression of circAFF1 decreased the levels of miR-516b (Figure 4G). These results implied that circAFF1 might function as a competing endogenous RNA (ceRNA) through targeting miR-516b.

### miR-516b Counteracts the Inhibitory Effect of Hypoxia on Vascular Endothelial Cells

**FIGURE 5:**
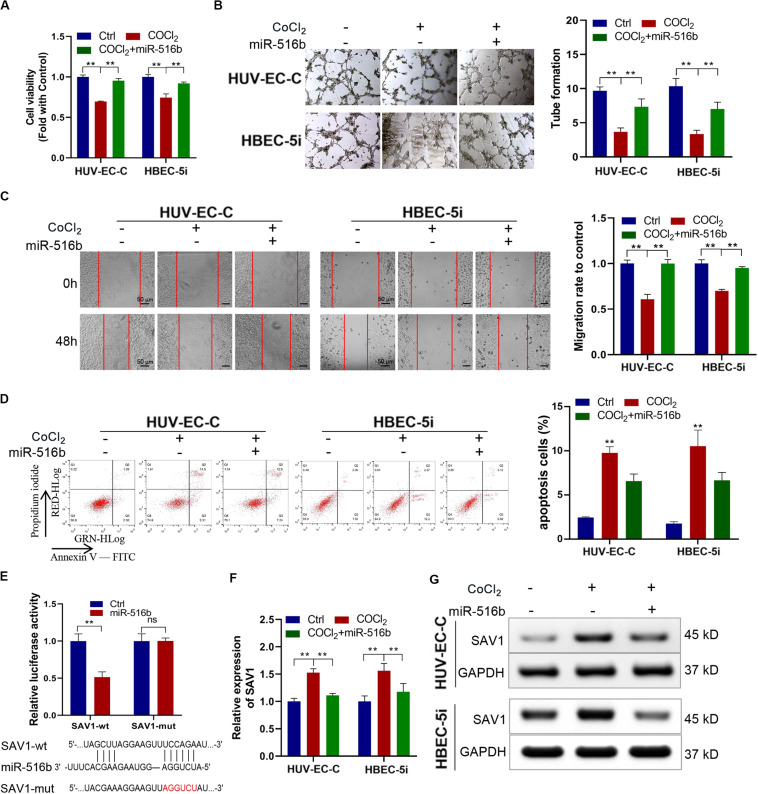
Under CoCl_2_ treatment, upregulated miR-516b restored the proliferation and tube formation and inhibits the apoptosis of endothelial cells by targeting SAV1. **(A)** The proliferation ability of HUV-EC-C and HBEC-5i cells transfected with miR-516b mimics was evaluated by CCK-8 assay. **(B)** The tube formation capability of HUV-EC-C and HBEC-5i cells transfected with miR-516b mimics was evaluated. **(C)** The migration capability of HUV-EC-C and HBEC-5i cells transfected with miR-516b mimics was evaluated by wound healing assays. **(D)** The apoptosis capability of HUV-EC-C and HBEC-5i cells transfected with miR-516b mimics was evaluated by apoptosis assay. **(E)** Schematic of SAV1 wild-type (wt) and mutant (mut) luciferase reporter vectors and Luciferase reporter assay in HEK293T cells co-transfected with SAV1-wt or SAV1-mut plasmids. **(F,G)** Under hypoxic conditions, qRT-PCR and Western blot analysis detected SAV1 in HUV-EC-C and HBEC-5i cells after being transfected with miR-516b mimics. Data are presented as means of three experiments, and error bars represent SD (**P < 0.01).

In order to verify the function of miR-516b, we overexpressed miR-516b in HUV-EC-C and HBEC-5i cells with hypoxia. After detecting the expression of miR-516b by PCR, we used tube formation, wound healing and apoptosis experiments to test the reversal effect of miR-516b on vascular endothelial cell function after hypoxia. Results showed that, the miR-516b mimics could reverse the inhibitory effect of hypoxia on the proliferation (Figure 5A), tube formation (Figure 5B) and migration (Figure 5C) but inhibit the apoptosis (Figure 5D) of HUV-EC-C and HBEC-5i cells. According to miRBase prediction, miR-516b could target SAV1 3’untranslated region (3′UTR). To prove these findings, we conducted luciferase reporter assays using wild-type or mutant version of SAV1 3′UTR. The miR−516b mimics repressed the luciferase activity of the wild−type reporter but not that of the mutant-type reporter (Figure 5E). Simultaneously, we evaluated the expression level of SAV1 in endothelial cells by transfecting miRNA mimics based on the association between SAV1 and miR-516b. The expression level of SAV1 was detected by qRT-PCR and Western blot, and results showed that SAV1 was obviously upregulated in HUV-EC-C and HBEC-5i cells under hypoxia (Figures 5F,G). Taken together, these data indicated that SAV1 is a target gene of miR−516b.

### SAV1 Inhibits Endothelial Cells Through YAP1 Phosphorylation

**FIGURE 6:**
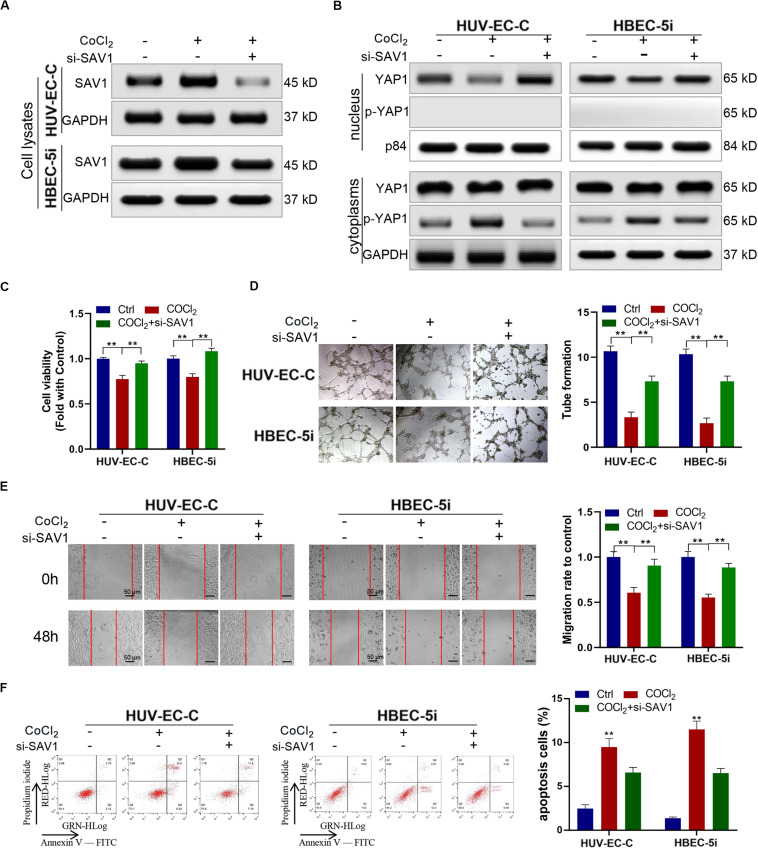
Downregulated SAV1 promoted endothelial cells progression through YAP1 activation. **(A,B)** Under CoCl2 treatment, Western blot analysis of SAV1, YAP1, and p-YAP1 was detected in HUV-EC-C and HBEC-5i cells after silencing SAV1. **(C)** Under CoCl_2_ treatment, the proliferation capability of HUV-EC-C and HBEC-5i cells after silencing SAV1 was evaluated by CCK-8 assays. **(D)** Under CoCl_2_ treatment, the tube formation capability of HUV-EC-C and HBEC-5i cells after silencing SAV1 was evaluated. **(E)** Under CoCl_2_ treatment, the migration capability of HUV-EC-C and HBEC-5i cells after silencing SAV1 was evaluated by wound healing assays. **(F)** Under CoCl_2_ treatment, the apoptosis capability of HUV-EC-C and HBEC-5i cells after silencing SAV1 was evaluated by apoptosis assay. Data are presented as means of three experiments, and error bars represent SD (**P < 0.01).

We downregulated the expression SAV1 in HUV-EC-C and HBEC-5i cells with SAV1 siRNA and then evaluated the expression level of SAV1 in HUV-EC-C and HBEC-5i cells by Western blot (Figure 6A). As a key protein of the Hippo/YAP pathway, SAV1 can phosphorylate YAP1 and prevent it from entering the nucleus. The results here showed that SAV1 could indeed cause up-regulation of SAV1 and YAP1 phosphorylation under hypoxic conditions, while knockdown of SAV1 could down-regulate YAP1 phosphorylation and promote its nucleation (Figure 6B). We also assessed the functional effect of SAV1 in HUV-EC-C and HBEC-5i cells. As expected, under hypoxic conditions, SAV1 downregulation significantly promoted the proliferation (Figure 6C), tube formation (Figure 6D), migration (Figure 6E) and inhibited the apoptosis (Figure 6F) of HUV-EC-C and HBEC-5i cells.

### miR-516b Abolishes the Inhibitory Role of circAFF1 in Endothelial Cells

**FIGURE 7:**
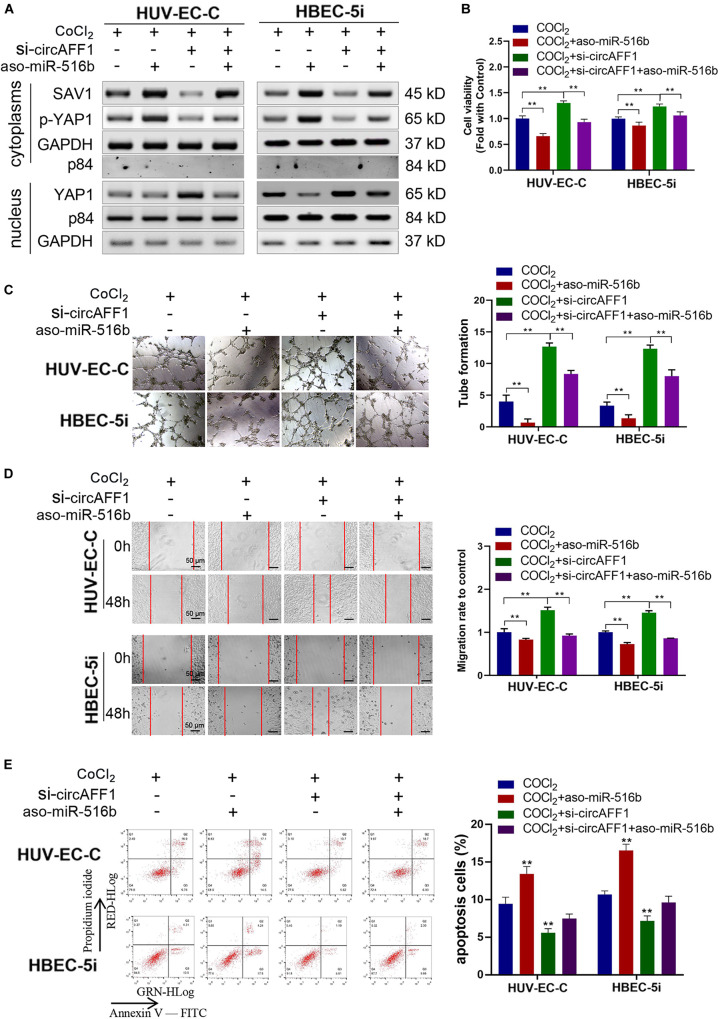
miR-516b reversed the effect of circAFF1 on endothelial cells. **(A)** Western blot analysis demonstrated that circAFF1 can counteract the influence of miR-516b mimics on SAV1, YAP1, and p-YAP1 expression in HUV-EC-C and HBEC-5i cells. **(B)** CCK-8 assay indicated that the proliferation ability of HUV-EC-C and HBEC-5i cells transfected with aso-miR-516b was reversed when co-transfected with si-circAFF1. **(C)** Tube formation ability of HUV-EC-C and HBEC-5i cells transfected with aso-miR-516b was reversed when co-transfected with si-circAFF1. **(D)** Wound healing assays indicated that the migration capability of HUV-EC-C and HBEC-5i cells transfected with aso-miR-516b was reversed when co-transfected with si-circAFF1. **(E)** Apoptosis assay indicated that the apoptosis ability of HUV-EC-C and HBEC-5i cells transfected with aso-miR-516b was reversed when co-transfected with si-circAFF1. Data are presented as means of three experiments, and error bars represent SD (**P < 0.01).

Rescue experiments were performed under hypoxic conditions by co-transfecting si-circAFF1 and aso-miR-516b in endothelial cells to estimate whether the effects of circAFF1 on endothelial cells by sponging miR-516b. Western blot assay indicated that, compared with the HUV-EC-C and HBEC-5i cells transfected only with aso-miR-516b, the SAV1 and p-YAP1 protein levels were partly increased, and YAP1 was obviously decreased in the HUV-EC-C and HBEC-5i cells co-transfected with si-circAFF1 and aso-miR-516b (Figure 7A). Meanwhile, the proliferation (Figure 7B), tube formation (Figure 7C), migration (Figure 7D) of the HUV-EC-C and HBEC-5i cells co-transfected with si-circAFF1 and aso-miR-516b were increased, and cell apoptosis (Figure 7E) was reduced compared with those of the HUV-EC-C and HBEC-5i cells transfected only with aso-miR-516b, suggesting that circAFF1 downregulation can partly abolish the inhibition of proliferation, tube formation, migration and invasion induced by miR-516b. Similarly, circAFF1 downregulation can also partly decrease the miR-516b-mediated apoptosis of endothelial cells. Collectively, these results demonstrated that circAFF1 inhibited the progression of endothelial cells under hypoxic conditions partly through miR-516b.

### circAFF1 Was Upregulated in Patients With Subarachnoid Hemorrhage

**FIGURE 8:**
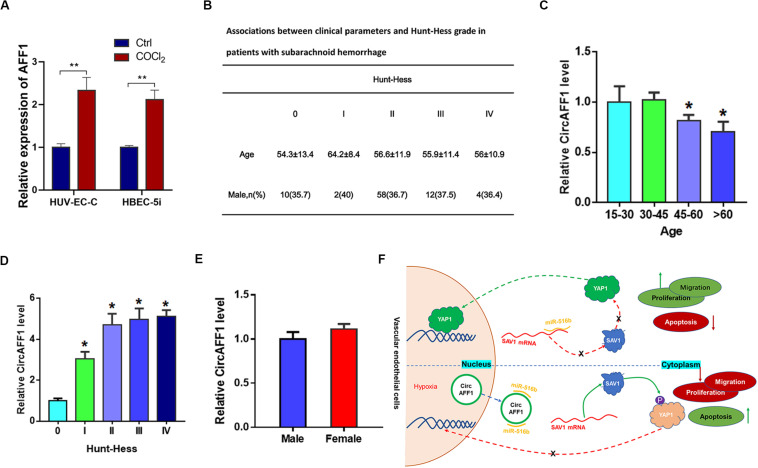
Expression of circAFF1 in patients with subarachnoid hemorrhage. **(A)** CircAFF1 levels in exosomes derived from HUV-EC-C and HBEC-5i cells after CoCl2 treatment. **(B)** Clinical information of 235 subarachnoid hemorrhage patients. **(C)** cirAFF1 expression in patients at different ages. **(D)** Expression of cirAFF1 in patients with different Hunt-Hess grades. **(E)** Expression of cirAFF1 in patients with different genders. **(F)** Mechanism of vascular endothelial cell dysfunction caused by circAFF1 induced by hypoxia. Data are presented as means of three experiments, and error bars represent SD (*P < 0.05 and **P < 0.01).

The above results show that hypoxic environment will cause abnormal function of vascular endothelial cells and increase of circAFF1. We speculate that circAFF1 may be used as a diagnostic marker for hemorrhagic vascular diseases, such as SAH. In order to determine this effect of circAFF1, we first tested whether the endothelial cells treated with CoCl2 would secrete more circAFF1 out of the cell through the exosome pathway. The results showed that there was indeed more cricAFF1 in endothelial-derived exosomes after CoCl2 treatment (Figure 8A). The occurrence of SAH is closely related to vascular dysfunction. We collected serum from 235 patients with subarachnoid hemorrhage and classified them into 0–IV grades according to Hunt-Hess classification (Figure 8B). We analyzed the expression level of circAFF1 and found that the expression of circAFF1 was higher in patients older than 45 years old (Figure 8C) and patients with higher Hunt-Hess levels (Figure 8D). While, the expression of circAFF1 was not obviously correlated with gender (Figure 8E). This suggests that the upregulation of circAFF1 caused by hypoxia and subsequent vascular endothelial injury may be used for the early diagnosis of potential vascular-related diseases (Figure 8F).

## Discussion

Accumulating evidence indicates that circRNAs might play critical roles in the development and progression of different disease (Liu et al., 2019; Su et al., 2019). Previous studies revealed that circRNAs are miRNA sponges that exert various biological roles. The cytoplasmic localization of circRNA is closely associated with miRNA sponging (Hall et al., 2019). As the most well-known circRNA, ciRS-7 contains multiple miR-7 binding sites and decreases the biological effect of miR-7 on its target genes via sponging miR-7 (Hansen et al., 2013; Hanniford et al., 2020). In the present study, we found that circAFF1 was upregulated in endothelial cells that under hypoxic conditions, which is consistent with the findings of Boeckel JN (Boeckel et al., 2015). Ectopic expression of circAFF1 can suppress the proliferation and tube formation and promote the apoptosis of endothelial cells *in vitro*. SiRNA-mediated silencing of circAFF1 significantly promoted the proliferation, tube formation, migration and invasion and suppressed the apoptosis of endothelial cells. Through bioinformatics analysis, biotinylated RNA pull-down, and dual-luciferase reporter assays, we identified miR-516b as a target of circAFF1. Furthermore, miR-516b can reverse the effects of circAFF1 on endothelial cells. These results suggested that circAFF1 could serve as a miRNA sponge for miR-516b and inhibit the proliferation and tube formation and promote apoptosis of endothelial cells.

Hippo is a conserved signaling pathway consisting of a serine kinase cascade and is a key regulator of cell proliferation and organogenesis (Hong et al., 2016; Sun and Irvine, 2016). YAP1 and TAZ are the final effectors of the Hippo signaling pathway. When YAP1 and TAZ are dephosphorylated, they enter the nucleus to act as transcription cofactors and participate in the occurrence and development of various diseases. However, the phosphorylation modification will block its entry into the nucleus and thereby inhibit cell function (Plouffe et al., 2015; Rausch and Hansen, 2020). As a core component of Hippo signaling pathway, SAV1 can promote the phosphorylation of YAP1 and participate in a series of cell function regulation, including vascular endothelial cells (Kim et al., 2017). In the present study, we found that SAV1 was a directly target gene of miR-516b. We found that hypoxic conditions induced up-regulation of circAFF1 and down-regulation of miR-516b in endothelial cells, which lead to the formation of more SAV1 proteins and subsequently caused the phosphorylation of YAP1. Phosphorylation of YAP1 then prevented it from entering the nucleus and eventually impaired the function of vascular endothelial cells. Accumulating evidence have shown that hypoxia can induce vasodilation and vascular leak (Dieterich et al., 2006; Eckle et al., 2008; Wong et al., 2017), which may cause bleeding or bleeding, especially during the birth of the newborn. In tumor, Hypoxic lung cancer cells increase vascular permeability and cancer trans-endothelial migration by secreting exosomes (28436951). Here, we found abnormally high levels of circAFF1 in the serum of SAH patients. This may be related to abnormal blood vessel function and increased permeability caused by hypoxia. In subsequent experiments, we will verify the diagnosis of abnormal expression of circRNA and evaluate it as a potential therapeutic target in multiple vascular function-related diseases. Examples include subarachnoid hemorrhage, cerebral infarction, myocardial infarction, and diabetes.

In summary, we found that hypoxia can induce the abnormal expression of circAFF1 in vascular endothelial cells, and it can increase the phosphorylation level and retention of cytoplasm of YAP1 by adsorbing miR-516b, and finally inhibit the normal proliferation of vascular endothelial cells and development. These hints that hypoxia can cause abnormal function of vascular endothelium, which will affect the normal development of the organ and the progress of the disease. Early detection of vascular function may prevent or control the development of related diseases.

## Data Availability Statement

The raw data supporting the conclusions of this article will be made available by the authors, without undue reservation, to any qualified researcher.

## Ethics Statement

The studies involving human participants were reviewed and approved by Tianjin Huanhu Hostital. The patients/participants provided their written informed consent to participate in this study. Written, informed consent was obtained from the individuals for the publication of any potentially identifiable images or data included in this article.

## Author Contributions

XT, YF, SC, and HGW conceived and designed the experiments. HY, DM, ML, ZZ, and XY performed all the experiments. HY, CW, BL, XL, KL, and JB analyzed the data. HY wrote the manuscript. HZ revised the manuscript. All authors read and approved the final manuscript.

## Conflict of Interest

The authors declare that the research was conducted in the absence of any commercial or financial relationships that could be construed as a potential conflict of interest.

## Footnotes

1
http://circrna.org/

## Abbreviations

AKT1: AKT serine/threonine kinase 1
CCK-8: cell counting Kit-8
CDK2: cyclin-dependent kinase 2
cDNA: complementary DNA
CI: cerebral infarction
circRNAs: circular RNAs
Ctrl: Control
FBS: fetal bovine serum
FISH: Fluorescence *in situ* hybridization
miRNA: microRNA
OD: optical density
PDK1: pyruvate dehydrogenase kinase 1
PI: propidium iodide
qRT-PCR: quantitative real-time polymerase chain reaction
SAV1: salvador family WW domain containing protein 1
SD: standard deviation
SDS–PAGE: Sodium dodecyl sulfate-Polyacrylamide gel electrophoresis
siRNA: small interfer RNA
tRNA: tranfer ribonucleic acid
WT: wild type
YAP1: Yes-associated protein 1.
