# Corrigendum: CircAFF1 aggravates vascular endothelial cell dysfunction mediated by miR-516b/SAV1/YAP1 axis

**DOI:** 10.3389/fphys.2022.881750

**Published:** 2022-07-22

**Authors:** Hong-guang Wang, Hua Yan, Chen Wang, Mi-mi Li, Xin-ze Lv, Hai-dong Wu, Zhan-hai Fang, Dong-li Mo, Zhi-yuan Zhang, Bin Liang, Ke-guan Lai, Jing-yu Bao, Xue-jia Yang, Hong-juan Zhao, Shuang Chen, Yi-mu Fan, Xiao-guang Tong

**Affiliations:** ^1^ College of Pharmacy, Nankai University, Tianjin, China; ^2^ Department of Neurosurgery, Tianjin Huanhu Hospital, Tianjin, China; ^3^ Department of Neurology, Tianjin Huanhu Hospital, Tianjin Key Laboratory of Cerebral Vascular and Neurodegenerative Diseases, Tianjin, China; ^4^ Tianjin Institute, of Neurosurgery, Tianjin Huanhu Hospital, Tianjin, China; ^5^ Tianjin Key Laboratory of Early Druggability Evaluation of Innovative Drugs, Tianjin International Joint Academy of Biomedicine, Tianjin, China; ^6^ Drug Safety Evaluation Center, Tianjin International Joint Academy of Biomedicine, Tianjin, China; ^7^ Department of Neurosurgery, People’s Hospital of Ningxia Hui Autonomous Region, Yinchuan, China; ^8^ Department of Respiratory Medicine, Songjiang Sijing Hospital, Shanghai, China

**Keywords:** vascular endothelial cell, subarachnoid hemorrhage, hypoxic, circAFF1, YAP1

In the original article, there was a mistake in Figure 7C as published. In the tube formation results in Figure 7C, the CoCl_2_ group of HUVEC-C cells and the CoCl_2_/si-circAFF1/aso-miR-516b group of HBEC-5i misused the same picture. This is due to the similar naming of the two images**.** The correct Figure 7 appears below.

**FIGURE 7:**
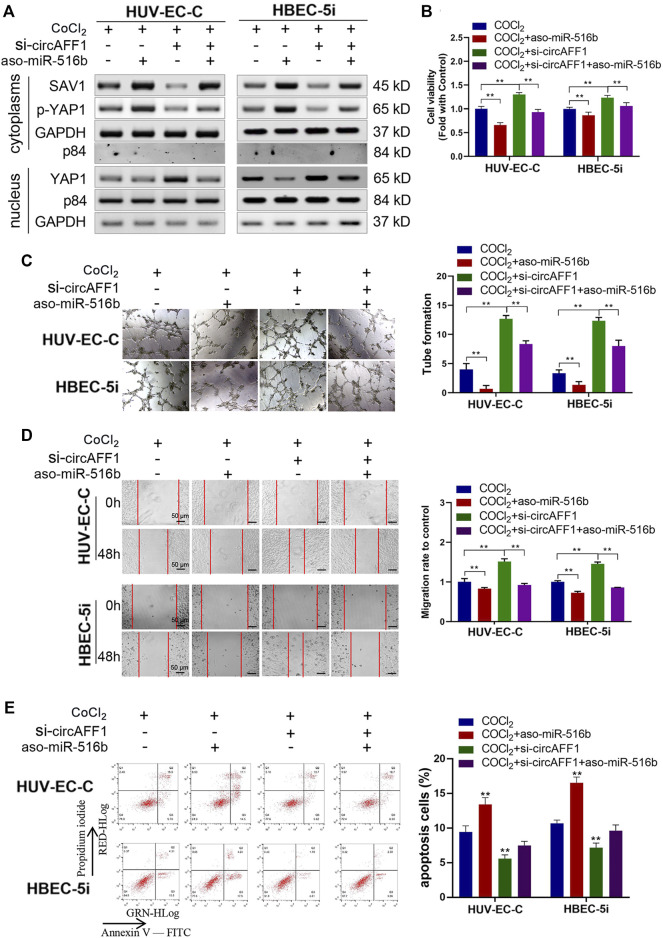
miR-516b reversed the effect of circAFF1 on endothelial cells. **(A)** Western blot analysis demonstrated that circAFF1 can counteract the influence of miR-516b mimics on SAV1, YAP1, and p-YAP1 expression in HUV-EC-C and HBEC-5i cells. **(B)** CCK-8 assay indicated that the proliferation ability of HUV-EC-C and HBEC-5i cells transfected with aso-miR-516b was reversed when co-transfected with si-circAFF1. **(C)** Tube formation ability of HUV-EC-C and HBEC-5i cells transfected with aso-miR-516b was reversed when co-transfected with si-circAFF1. **(D)** Wound healing assays indicated that the migration capability of HUV-EC-C and HBEC-5i cells transfected with aso-miR-516b was reversed when co-transfected with si-circAFF1. **(E)** Apoptosis assay indicated that the apoptosis ability of HUV-EC-C and HBEC-5i cells transfected with aso-miR-516b was reversed when co-transfected with si-circAFF1. Data are presented as means of three experiments, and error bars represent SD (^**^ *p* < 0.01).

The authors apologize for this error and state that this does not change the scientific conclusions of the article in any way. The original article has been updated.

